# Health IT Implementation and the Impact of the COVID-19 Pandemic on Clinician-IT Dynamics: Qualitative Study

**DOI:** 10.2196/57847

**Published:** 2025-02-11

**Authors:** Adeola Bamgboje-Ayodele, Adrian Boscolo, Mitchell Burger, Owen Hutchings, Miranda Shaw, Tim Shaw, Amina Tariq, Sundresan Naicker, Steven McPhail, Melissa Baysari

**Affiliations:** 1 Biomedical Informatics and Digital Health, School of Medical Sciences Faculty of Medicine and Health University of Sydney Camperdown Australia; 2 Sydney Local Health District Sydney Australia; 3 Australian Centre for Health Services Innovation and Centre for Healthcare Transformation Queensland University of Technology Brisbane Australia; 4 Digital Health and Informatics Directorate Metro South Health Brisbane Australia

**Keywords:** health IT, implementation, COVID-19 pandemic, process evaluation, sociotechnical factors, virtual hospital, COVID-19

## Abstract

**Background:**

The COVID-19 pandemic necessitated the rapid development and implementation of health ITs to support health care delivery. Health IT implementation is difficult at the best of times, due to complex sociotechnical challenges that vary across contexts and settings; however, it is currently unclear how the pandemic impacted health IT implementation processes. The aim of this study was to explore the impact of the pandemic on health IT implementation processes, including pre- and postimplementation phases, and identify the sociotechnical factors that shaped health IT implementation during an unprecedented circumstance.

**Objective:**

This study aimed to explore the impact of the pandemic on HIT implementation processes, including pre- and postimplementation phases, and identify the socio-technical factors that shaped health IT implementation during an unprecedented circumstance.

**Methods:**

Participants were from one of two teams: (1) health care staff members (doctors, nurses, nurse unit managers, and support staff members) from a virtual hospital in Australia; and (2) IT professionals within the broader health care organization assigned to the hospital. Participants took part in an interview or focus group from July to November 2022. Participants were asked to describe the process used for rapid health IT design and implementation during the COVID-19 pandemic. Qualitative data were analyzed thematically.

**Results:**

A total of 15 participants took part in the study. Both internal and external team structures, and the communication pathways that underpinned these, were reported to influence the health IT lifecycle, which in turn impacted outcomes, particularly when perceived normal ways of working were challenged during the pandemic. Across the pre-post lifecycle, preimplementation processes were viewed to be most impacted by the COVID-19 pandemic. Participants reported that their roles and responsibilities changed during health IT implementations in the pandemic, impacting co-design processes and highlighting the need for health IT implementation processes to cater for new work and the redistribution of existing work.

**Conclusions:**

Our study uncovered the negative impact of the COVID-19 pandemic on team structures, communication pathways, and health IT preimplementation processes (project management and co-design). While health care organizations are keen to transition beyond the ways of working during the pandemic, it is imperative to learn from the health IT implementation successes and failures that occurred in the pandemic via process evaluations. Our evaluation offers learnings for research (an adapted interdisciplinary team communication framework), practice (the need for health care organizations to review their communication structures, IT staff skills, and proposed processes), and education (the need for better education and training of IT professionals working in clinical settings on health concepts) on health IT implementations as the world transitions to the “new norm.”

## Introduction

The implementation of health IT to improve safety, quality, and efficiency in health systems is increasing globally, more so during the COVID-19 pandemic [1-3]. However, health IT implementations are notoriously difficult due to complex sociotechnical challenges, which vary across contexts and are often hard to navigate and envisage for those managing change [4]. Despite significant efforts to identify and apply the sociotechnical factors that enable successful health IT implementations, this remains an ongoing challenge [5]. One reason for this may be that the commonly used approach to evaluate existing health ITs tends to focus on assessing each aspect of the health IT implementation, rather than using a “whole of system” approach [6]. As new health IT implementations can learn from the evaluation of existing health ITs, there is a strong need for comprehensive evaluations that transcend a disease focus or preliminary adoption, to provide in-depth insights into the various sociotechnical factors that enable successful health IT implementations.

The Technology, People, Organizations, and Macro-environmental factors (TPOM) framework [5] is one model that provides a comprehensive understanding of the socio-technical factors impacting health IT implementations using a “whole of system” approach. TPOM has been used to evaluate a large-scale health IT implementation for the National Health Service [7] before the COVID-19 pandemic, and other health IT implementations in Europe and United States during the pandemic [8]. Unlike other health IT implementation frameworks [9], the TPOM framework is neither illness-specific, nor focused on the likelihood of adoption or spread, but considers both the microcontext of use and macroenvironmental dimensions that impact all stages of implementation [5]. Therefore, we use the TPOM framework as a lens to consider the gaps in evidence regarding the impact of the pandemic on key sociotechnical factors, across the four TPOM domains, that shape the outcomes of health IT implementations.

From a technology use perspective (TPOM domain 1), diverse health systems have leveraged health ITs in response to the COVID-19 pandemic. This has included the development of new devices and applications for remote consultations to minimize transmission of infection, and mobile apps to monitor vital signs data [1]. These health IT implementations experienced mixed success and highlighted that many technology-driven challenges (eg, data sharing across systems) present in the prepandemic times were perpetuated, and potentially magnified, during the pandemic and postpandemic “new-normal” times [10]. Furthermore, these challenges may impact the pre-post-implementation lifecycle, including perpetuating interoperability challenges [10]. However, little is currently known about the impact of the pandemic on these technology-driven challenges across the pre-post implementation lifecycle.

With people at the core of health IT implementations (TPOM domain 2), effective stakeholder engagement including collaboration between clinicians and IT staff is key [11,12]. However, the divide that exists between these 2 groups, such as communication challenges and different viewpoints on system performance and usability, has been well-documented [13-16]. The health system’s response to the pandemic was not limited to clinical care delivery, and virtual stakeholder engagement sessions in the context of health IT implementation became increasingly common to support rapid policy changes and social distancing guidelines [17]. As such, the use of flexible locations enabled more efficient use of clinician time in supporting the design and implementation of health ITs [10,18]. While it may be likely that greater use of virtual sessions, rather than face-to-face sessions can exacerbate the clinician IT divide, the impact of virtual engagement on health IT implementations since the pandemic has not been evaluated.

Health service organizations (TPOM domain 3), in response to the COVID-19 pandemic, increasingly aimed to become “learning health systems” intent on learning from what worked and what did not work during pandemic-driven health IT implementations, in an effort to transition to the “new-normal” [19,20]. Despite this intention, there have been limited process evaluations of health IT implementations in real-world health service organizations that span across the pre-post implementation phases, particularly in virtual hospitals. Virtual hospitals substitute in-person consultations with telephone or video consultations and often include asynchronous data collection from the patient via survey tools with or without real-time remote monitoring [21].

The macroenvironmental context (TPOM domain 4) during the COVID-19 pandemic placed unprecedented time pressure on health IT implementations, and other pressures on health care services [1,22]. Yet, the impact of the COVID-19 pandemic on the technology, people, and organizations involved in health IT implementation has not been evaluated.

This study set out to address the identified gaps in literature and knowledge across the 4 TPOM domains highlighted above by interviewing stakeholders involved in rapid implementations of health IT during the pandemic. In particular, we aimed to explore the impact of the pandemic on health IT implementation processes, including pre- and postimplementation phases, and identify the sociotechnical factors that shaped health IT implementation, to guide future successful implementations of health IT.

## Methods

### Study Design

This was a cross-sectional qualitative study. As a qualitative study design is appropriate for providing insights into people’s experiences of a complex phenomenon or activity [23], we conducted semistructured interviews to explore participants’ experiences. The COREQ (Consolidated Criteria for Reporting Qualitative Research) checklist was used to report the study (Multimedia Appendix 1).

### Setting and Recruitment

This study was conducted at a virtual hospital in Australia from July to November 2022. The virtual hospital was equipped with care pods (workspaces) with videoconferencing, telephone facilities, an electronic medical record, and used remote monitoring tools such as wearables, mobile apps, and dashboards. The majority of the videoconferencing and remote monitoring tools were simultaneously implemented during the COVID-19 pandemic. Although multiple teams were involved in health IT implementation at the study site, this study focused on the interdisciplinary relationship and communication processes between clinicians and IT teams. Both teams are part of the same health service but not colocated within the same building.

Using purposive sampling, all clinical (ie, doctors, nurses, and nurse unit managers) and nonclinical staff members from the virtual hospital were invited by email to participate in interviews based on their involvement in health IT implementations, as identified by a clinician-researcher team member who works at the virtual hospital. Participants were eligible for purposive sampling if they were (1) either doctors, nurses, nurse unit managers, or nonclinical support staff members, and (2) took part in any pre or postimplementation activity in health IT implementations at the virtual hospital since the hospital’s establishment in March 2020. We conducted semistructured interviews and a focus group discussion to collect qualitative data. Due to scheduling constraints and the limited time availability of participants, we conducted a focus group discussion when individual interviews were not possible.

### Data Collection Procedure

The interview questions were broadly guided by the TPOM framework (Multimedia Appendix 2). Participants were asked to describe the process used for rapid health IT design and implementation during the COVID-19 pandemic and to reflect on aspects that worked well and those that did not. Except for one face-to-face interview, other interviews and the focus group were conducted online. For the focus group discussion, participants were fully informed about the data confidentiality responsibilities of both the researchers and the participants and the necessary housekeeping rules. The moderator (AB-A) asked open-ended questions throughout the session to allow participants to share information they were comfortable with and prompted those who spoke less frequently to contribute to the discussion to ensure inclusivity and diversity of voices using probing techniques, tracking of questions for completion, in line with existing research [24]. Data collection ended when no new information emerged, and saturation was achieved. All sessions were recorded and transcribed verbatim. The sessions were conducted by AB-A, a postdoctoral researcher experienced in qualitative research and health IT implementations, and she had no previous relationship with the participants.

### Data Analysis and Interpretation

Deidentified transcripts were thematically analyzed independently by 2 researchers (ABA and MB), who first identified and coded the transcribed data through inductive thematic analysis, using a data-driven approach to understand participants’ experiences and perceptions [25,26]. Following this, the researchers mapped the identified codes to the interdisciplinary team communication framework of Kuziemsky et al [27] for assessing interdisciplinary teams through the exploration of sociotechnical and other factors that drive teamwork. The framework by Kuziemsky et al [27] is based on Donabedian’s [28] meta-concepts of structures, processes, and outcomes, which posits that structures lead to processes, which lead to outcomes [28]. The framework by Kuziemsky et al [27] was selected to provide an understanding of the complex and nonlinear structures, processes, and outcomes involved in pandemic-driven health IT implementation as experienced by the 2 interdisciplinary teams (clinicians and IT staff).

Our analytical approach included data familiarization, coding, generating initial themes, reviewing potential themes, defining and naming themes, and producing the report [29]. Specifically, the 2 researchers (ABA and MB) familiarized themselves with the transcribed data by reading it multiple times before coding independently. After initial code generation, the researchers developed themes by merging codes with a shared meaning. Following this, the researchers reviewed the candidate themes to ensure coherent patterns were formed which contributed to the overall narrative and interpretation of the dataset. Finally, to ensure consistency and improve study rigor, the researchers discussed and reported the themes in relation to the dataset and the research aim. The 2 researchers met frequently throughout data collection and analysis to compare themes. Disagreement in themes was discussed until a consensus was reached. Emerging themes were presented to some participants and key stakeholders, and feedback received informed the final refinement of the themes.

### Ethical Considerations

Ethics approval was obtained from the hospital’s Human Research Ethics Committee (X21-0362 & 2021/ETH11708). Written informed consent was obtained from all participants, who were informed of their right to withdraw from the study at any time. The data presented in this article have been deidentified.

## Results

A total of 15 participants took part in this study, one more person was invited but was too busy to participate. Out of 8 staff at the virtual hospital, including representation from clinical and nonclinical roles, and 7 staff from the IT department (refer to Table 1 for demographic details). The mean duration of the interviews (n=11) was 58 (range 32-93) minutes and the focus group (n=4 participants) lasted 62 minutes.

Our findings are organized according to the meta-concepts of structures, processes, and outcomes. The concepts and subconcepts that emerged for each of these 3 meta-concepts are described using quotes from the data to illustrate examples of the concepts and subconcepts.

**Table 1:** Demographic characteristics.

Demographic characteristic		Values
**Role, n (%)**		
	Nurse	2 (13)
	Nurse unit manager	3 (20)
	Doctor	2 (13)
	Nonclinical	1 (7)
	IT professionals	7 (47)
**Team, n (%)**		
	Virtual hospital staff	8 (53)
	IT staff	7 (47)
**Years of experience, n (%)**		
	0-5	2 (13)
	6-10	4 (27)
	11-20	6 (40)
	>20	3 (20)
**Sex or sex assigned at birth, n (%)**		
	Male	4 (27)
	Female	11 (73)
Age (years), mean (range)		41 (28-62)

### Structure

**Figure 1:**
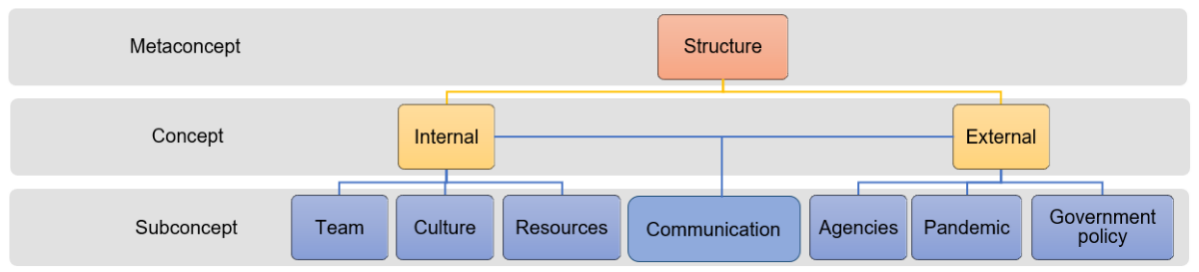
Ontology of concepts and sub-concepts for the structures of a health care organization during the COVID-19 pandemic.

Structure represents a meta-concept that describes how the virtual hospital organized their team, culture, and resources internally, and how it interacted with external entities via various communication pathways. Structure was therefore conceptualized as internal and external concepts with communication as a subconcept that enabled the relationship between the internal teams and external teams and agencies (Figure 1).

#### Internal Structure

Internal structure describes how the team functions from within. Out of 3 main subconcepts describing the internal structure emerged from the data. These were team, culture, and resources. Culture and resources were described in relation to dynamics within each of the teams (clinician and IT).

##### Culture Within Clinician Team

The clinician team comprised doctors, nurses, and nurse unit managers. Within the clinician team, participants reported a culture that fostered the ability of staff members to adapt to rapid changes that frequently occurred during the pandemic.

So everyone is working very collaboratively for things to be implemented efficiently, but also quickly …And I think it’s purely based on the fact that we got so used to it with the rapid changes with COVID. And we knew we had to adapt to the change very quickly.

P005, clinician team

Also, participants described strong leadership support for health IT implementations, particularly leadership willingness to receive and respond to staff members feedback.

I had a lot of experience working on the wards before this. And not much changed, really ever. You know, things really were kind of, this is the way it’s done [but here] I think, they [executives] really listen to the feedback of staff and take that on board.

P004, clinician team

##### Culture Within IT Team

Within the IT team, participants reported a learning culture, with IT members reflecting on, and constantly, improving health IT implementation processes.

now we’ve stepped back and gone, hang on, let’s just take some time to stop the crisis. Think about what our role is, educate [the clinician team], and give them a process that they can follow, and a process that can follow quite easily…Yes, we have that process now.

P010, IT team

##### Resources Within IT Team

Regarding operational resources, the IT team perceived that the clinician team appeared to have insufficient time to participate in the co-design of health ITs, although it was acknowledged that pandemic patient care is, and should be, their priority. For example, a participant said:

…the problem is time, they [clinician team] never have enough time.

P010, IT team

High staff turnover within the IT team was identified by participants as another resource concern impacting health IT implementations.

And we had people coming and going, so we had business analysts, stop starting. And then we had a six week period where we didn’t have a business analyst and we were trying to develop workflow and testing and all of those things… it was a new project team from our side.

Focus group, P013, IT team

#### External Structure

External structure describes the influence of external agencies and factors on health IT implementation. Out of 3 influences were identified including the COVID-19 pandemic, vendors, and government policies.

##### COVID-19

The onset of the COVID-19 pandemic necessitated the rapid development of virtual health care services to help manage the large numbers of patients affected by the disease and mitigate the risk of shortage of hospital resources. Participants explained that this led to rapid COVID-19 management policy changes, but also unrealistic delivery timelines. The impact was seen to be rapid design without time for adequate reflection.

remember policy were changed and we have to roll it out within the week. So we were given one week to do [it]…which caused a lot of problems, because you didn’t have time to think about downstream implications. And you really didn’t have time to think about, you know what, there’s good design required for this. It was just, ‘here is what you need to meet, go on ahead and meet that’. So the design was very, very rapid. And it allowed no time to really review.

P010, IT team

##### Vendors

In the context of a virtual hospital where a variety of remote monitoring tools were rapidly and simultaneously implemented during the COVID-19 pandemic, internal teams said that they engaged with multiple vendors to deliver the required technologies. However, participants from the IT team reported that they were unable to themselves configure certain vendor products, which meant that simple configuration tasks were delayed. For example, a participant said:

Everything has to go back to the vendor. But that’s also one of the features that we wanted to ask them that we could have is to for us to basically be able to configure it ourselves.

P009, IT team

##### Government Policy

Furthermore, it was perceived that the sustainability of the virtual hospital depended on the availability of supportive legislative policies. Participants reported that there was a need for policies to change to recognize virtual hospitals as a viable hospital system.

…a lot of systems are built around legislation. And the change in those legislations then mean, we’re able to do what we want to do. Because at the moment, the definition of a hospital is not what we are in terms of what we want to do.

P003, clinician team

##### Communication

This is a subconcept that enables the relationship within internal teams and between internal and external teams or agencies for example, state government agencies, vendors so on. Themes emerged surrounding the communication medium and communication approach.

##### Navigating the Virtual

In terms of the communication medium, virtual co-design sessions became increasingly common during the COVID-19 pandemic to support the rapid design and development of health ITs under time pressure. However, participants explained that virtual co-design sessions often removed the “human” from “human-centered design.” Face-to-face co-design sessions were preferred over remote sessions because it was easier to understand each person’s role in the project, have informal conversations, and build a social connection.

…meetings are online, everything is via [online meeting platform], and that physical element is missing. And it’s sort of, you know, watered down the prototyping and the UX [user experience] bit…. Yeah, you don’t hear that hallway conversation. … And without these hallway conversations, it seems like yeah, there’s no collaboration that happens.

P009, IT team

Some participants described the virtual sessions as transactional and rapid, impacting the interpersonal relationships between project team members as well as the project.

A lot of the other projects that we do, we have team forming activities, we get to know each other…We missed all that because there was no time to do it.

P010, IT team

##### Varied Lexicon

Regarding the communication approach within internal teams, participants from both parties reported that clinicians had limited IT literacy and IT teams had limited health literacy, which resulted in miscommunication and key messages being lost in translation. For example:

So technically they use English words, and I use English words, but we have different interpretations of what those words mean.

P002, clinician team

Another participant said that:

we just tell ICT [information and communications technology] and ICT will consult with [the vendor] … and sometimes our request gets lost in translation.

P005, clinician team

In addition, participants from the IT team acknowledged that it was important for their team members to simplify complex language and tailor communication to individuals when communicating with clinicians.

…like, [staff name]’s great, but [they] use very, very complex language. And sometimes we get lost in that. And so I think that just makes [clinician team] just turn off. And so having to rephrase things all the time, just so they understand is a problem. I think we need to make it simpler, like the development process.

P010, IT team

##### Bridging the Transparency Gap

When reflecting on the communication approach between the internal and external teams, participants from the IT team reported limited transparency and responsiveness from external teams, regarding timelines, strategy, and the continuous improvement of the technology. For example:

…there were a lot of problems with [external party] committing to things, also being transparent, showing what they’re up to, showing what their intentions were in terms of timeframes in terms of strategy, where the product will be going.

P011, IT team

### Processes

**Figure 2:**
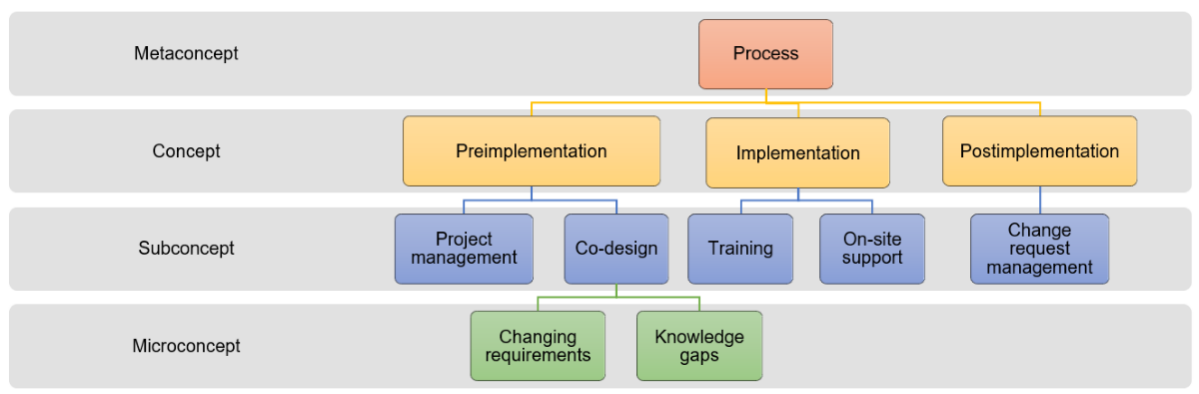
Ontology of concepts and sub-concepts for health IT implementation processes during the COVID-19 pandemic.

In this study, Process refers to the health IT development lifecycle and was conceptualized as preimplementation, implementation, and postimplementation phases, as shown in Figure 2.

#### Preimplementation Processes

Preimplementation processes were reported to involve activities undertaken when planning and co-designing HITs. During preimplementation, participants discussed issues related to both project management and co-design.

##### Optimizing Project Management

In terms of project management, participants raised concerns about how project meetings were planned, managed and documented. For example, regarding time management 1 participant said:

Project status meetings with clinicians, we schedule 30 minutes just to say these are the quick updates, but then it goes on for like an hour... I feel like sometimes we don’t respect their [clinician team] times in terms of a project team perspective.

P009, IT team

Another participant explained that meetings were not well documented because they were conducted virtually, and the minutes were generated electronically:

…there were no minutes for almost all the meetings. And then the same questions would come up three months later. And we’d say, well, we discussed this in February. Can we go back to that meeting? No. They’re all recorded on [videoconferencing platform], I hate. I hate meetings recorded on teams. That is not minutes. That does not give people an opportunity to reflect on what was said and see if it is what they said.

P002, clinician team

##### Adapting Co-Design to Shifting Realities

Regarding the co-design process, participants described challenges associated with changing requirements over time, and knowledge gaps within both the clinician and IT team.

##### Adapting to Changing Requirements

Participants reported that fluctuations in COVID-19 patient numbers in different waves of the pandemic led to changes in the system requirements gathered over time:

So it was started 12 months prior, and then stopped, and then came into fruition or asked to be activated, once the real COVID number started coming through in that sort of mid-year … So the requirements that were gathered 12 months prior were somewhat different to the requirements that were needed for the COVID dashboard proper, because it had changed.

P012, IT team, Focus group

Furthermore, participants stated that the pandemic had placed unprecedented pressure on IT teams resulting in a change in their roles and responsibilities. For example:

…the pandemic has put a lot of pressure or more pressure on ICT services staff because you now need to learn a lot more and really quickly to be able to deliver digital solutions not just for clinicians, now it has moved on to patients and now you have to learn AI, clinical decision support systems and all of that. And at a fast pace.

P009, IT team

Participants explained that rapid upskilling was needed with user experience (UX) design one of the core skills necessary in the new health IT implementation landscape:

…we’re not in that UX [user experience] business … we usually just configure things based on what our suppliers have…we don’t have that core capability.

P009, IT team

##### Bridging the Knowledge Divide

In addition, some participants reported that the clinician team had knowledge gaps regarding the system development processes, which influenced their expectations around the levels of complexity of some technology requirements and delivery timelines. For example, a participant said:

I think their expectations around time to deliver were very short…they don’t actually quite understand… that, you’ve got to do a build, you’ve got to put it into a non-production environment, you’ve got to validate it, it’s got to go into a staging environment, it's got to be tested, and then it's’got to go into production’environment. And if it's a major change there might be security implications, so there’s this whole group of people that are involved, there’s an integration activity.

P010, IT team

Participants within the clinician team also mentioned that they were unclear about their level of involvement in the co-design process and the level of technical literacy that was required:

if I’d known how much work this would be, and how technical it became … I never would have put my hand up to build something. I thought I was just providing some clinical advice about how we use it.

P002, clinician team

Conversely, some participants reported that the IT team had knowledge gaps regarding clinical staff roles and levels of seniority, which made it challenging to know when the IT team could engage clinicians; which clinicians should be engaged; and at what stage in the co-design process the clinicians should be engaged. For example, a participant said:

So I got asked the other day by the project lead what the difference between a consultant and a registrar is... And for us. That’s a really important distinction. And we’ve worked together for 12 months. And [they] didn’t know that difference.

P002, clinician team

In addition, participants within the IT team stated that their approach to co-design was not tailored to user needs and rarely included a visual of the user design preferences, noting this to be a knowledge gap within the team.

So I think there’s a really low level of prototyping or co-design that’s happening. ... because people are not comfortable with that level of creating visuals for people… And I guess UX people don’t only do visual design, but they try to understand the problems more. So I think we lack a lot in that first part of first understanding people and understanding what they need.

P009 IT team

#### Implementation Processes

Implementation processes involved activities undertaken to facilitate the successful rollout and uptake of health IT. Two main subconcepts describing implementation processes emerged from the data: Training and on-site support.

##### Tailored Training Strategies

Participants generally viewed training positively and explained that training provided by educators within the clinician team had been tailored for each model of care and was excellent. For example, a participant stated that:

Every time there’s a new or something goes live, then the staff, the nursing and the medical staff will get training in what are you supposed to do, how are you supposed to assess patients, And they get access to … instructions on what do you do with this patient as part of this trial? …So it’s a bit different with each different ones.

P001, clinician team

However, some participants identified a need to simplify training to accommodate those who are not technology-savvy and incorporate a more inclusive training approach for older staff.

Like, it’ll be this huge mass of information. And then there’ll be this really essential thing. And I’ll think I didn’t know that. And then I look at my notes. And I think, why didn’t I know that? And I look at my notes, and I didn’t know it, because nobody told it to me… I want something I can write on, like, I want something I can look at, because I’m getting older now, and even looking at a screen, I can’t see everything, you know, with these presentations. So for me to like strain, look and write, it’s just messy.

P007, clinician team

##### Defining Limits to On-Site Support

During the go-live period, participants from the clinician team reported that the length of time that on-site support was received from the IT team was insufficient. Participants explained that support was available for a predefined length of time, regardless of how many patients were seen, which was problematic when patient numbers were low:

So once we went live, we had a small group ICT team that was for support ... That support was supposed to run for 2 weeks. We haven’t got [any] patient [for the diverticulitis model of care] for around 3 weeks. So at the end of the 2 weeks. They were like hey guys, so our [diverticulitis model of care] support team is going to finish now. I’m like, we haven’t even had our 1st patient. So we don’t even know what type of support we will need.

P005, clinician team

#### Postimplementation Processes: Improving Vendor Communication

Postimplementation processes involved activities undertaken to maintain the health IT.

During postimplementation, participants identified one key process that had been challenging, that of issuing requests to the vendor to make technology changes. The change request process, where the IT team requested changes from the vendor, was viewed as ambiguous as it was unclear what tasks required formal requests for change and the associated cost implications. For example, a participant said:

So you have to raise change request for that, or you have to raise change requests, everything you want to do, we have to raise change requests for and then they don’t tell you if you actually have to pay for the change requested or not. …So they may be doing it for free but they don’t tell you that upfront, you only find out afterwards, it makes it really difficult to negotiate anything with them.

P011, IT team

Participants also emphasized that it was imperative to involve the IT team in the vendor product release cycle, to allow IT staff to plan and communicate in a timely manner with the stakeholders involved (eg, the clinician team). For example, a participant mentioned that:

…if you know there’s a bug fix for something coming up, then you can actually communicate that and say, yep, we’re on it, rather than keep asking, can we get the colour changed … knowing that in the third quarter release that the colours are all changing.

P013, IT team

Focus group Another participant said:

And then you can manage your stakeholders better ... And then have a co-design with the [vendor] team on what that product release cycle would look like as well. You know, having input in that.

P012, IT team, Focus group

### Outcomes

**Figure 3:**
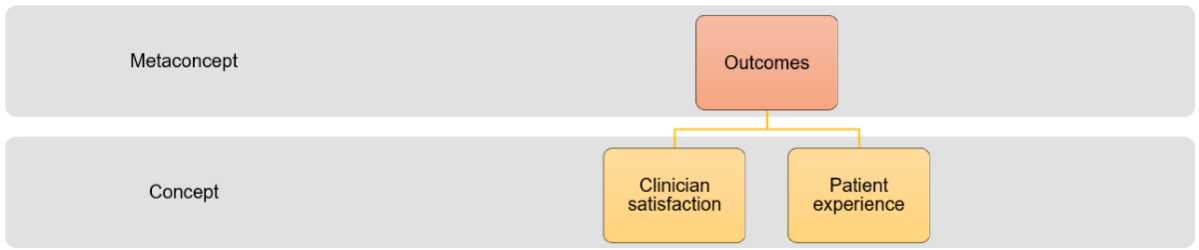
Ontology of concepts for the outcomes of health IT implementation during the COVID-19 pandemic.

When asked about the performance of the health IT that resulted from the rapid implementation process, participants described impacts on clinician satisfaction and patient experience (Figure 3).

### Clinician Satisfaction

Following health IT implementation at the virtual hospital, clinicians reported mixed levels of satisfaction with the technology, but they explained that this improved over time as teething issues with the technology were addressed. Participants raised concerns about negative UX, poor reporting capabilities of the technology, and limited system integration, with the majority of these problems being identified in the first few months following implementation. For example, a participant described a usability problem with one of the dashboards in use:

Another issue surrounding alerts is the fact that the dashboard is so big. You need to press… to see more on the dashboard… For us, it’s not a scroll. It’s a press, it says right here, you have to press it to go all the way to right now… if you don’t press all the way towards the right, you can only see half the heart rate.

P005, clinician team

Another participant stated that the initial reporting capability did not meet the needs of medical staff members:

I actually refused to move the medical workflow to it [dashboard] for two months, because there was no reporting. So, you know, they moved the nursing workflow, and we had medical workflow running on our teams, spreadsheets, and I was like… there’s no way of me telling which doctor was asked to see which patient at what time.

P002, clinician team

### Perceived Patient Experience

As the IT team received direct reports of patient experiences with the remote monitoring mobile phone applications, participants described challenges related to the interface and customizability of the technology. For example, 1 participant said that:

Patient facing app…I don’t think it’s good. I think it doesn’t meet the need of the patient…It’s interface is terrible. The workflow is not very good. The customization is very, very difficult. And the timeframe for development is way too long for what we want to do.

P010, IT team

Also, another participant commented on the ability of the app to present personalized self-management information to patients:

Or even just have a link to the website that gives them information about like, heart health or something. They can’t do that. So it’s like, that’s not performing well in terms of the patient’s experience, because there’s an expectation for you. If you’ve downloaded an app, you expect more also the things to be contained in that app or will link you to things. Very simple feature set. I don’t think the patients are getting that.

P009, IT team

## Discussion

### Principal Findings

In this study, we found that both internal and external team structures and the communication pathways that underpinned these influenced the health IT lifecycle, and pre-to-post implementation, which in turn impacted outcomes, particularly when the normal ways of working were challenged during the pandemic. Preimplementation processes, particularly project management and co-design, were viewed to be most impacted by the COVID-19 pandemic. Specifically, as meetings were held virtually due to social distancing guidelines, and health IT implementations in the pandemic were rapid, participants reported that meeting documentation was automated as it was faster but the outcome of this approach was deemed suboptimal. Also, fluctuations in COVID-19 patient numbers in different waves of the pandemic led to changes in the system requirements gathered over time which led to the need to adapt to shifting realities during co-design. Taken together, our study brings to the fore, the knotty relationship between technology, people, organizational and macroenviromental factors across different health IT implementation phases, aligning with previous research [4].

### Comparison With Previous Studies

Effective interdisciplinary communication across the implementation lifecycle was key for successful health IT implementation during the pandemic. Our findings align with existing research demonstrating communication challenges between clinicians and IT teams prepandemic [13,14,30], but showed communication difficulties were exacerbated by time pressures and social distancing guidelines that arose during the pandemic. Our study revealed that the social fabric (ie, web of interactions and connections) that existed within interdisciplinary teams were disrupted due to the urgent need for responsiveness between the internal and external teams, changing system requirements, the communication medium and the varied lexicon between the 2 internal teams. This led to miscommunication, information being lost in translation, and poor interpersonal relationships. While our study adds to the growing body of knowledge on the clinician IT divide, it also highlights effective communication as a necessity for bridging the divide between clinicians and IT experts in time pressured contexts [30]. As the new norm is established, with longer health IT delivery timelines, more stable system requirements, and time for manual meeting documentation and review; factors contributing to miscommunication are minimized but not eliminated as issues such as the varied lexicon between the clinician and IT teams appear enduring.

Regarding the communication medium between teams during health IT implementations, our findings suggest the need to minimize virtual co-design sessions when possible. Virtual co-design sessions became increasingly common during the COVID-19 pandemic to support rapid policy changes and needs, which often occurred with limited experience, preparation or preference of the participants in those sessions. However, it allowed flexibility in the location of co-design and more efficient use of clinician time [18]. Despite these benefits, our interviews revealed that there was limited social connection within co-design teams which contributed to poor interpersonal relationships. This result aligns with that of a simulation study that experimentally manipulated an interdisciplinary co-design process and found that physical separation during co-design led to less social connectedness and misalignment in design outcomes between the subgroups [31]. Another study on virtual co-design with children during the pandemic found that sustaining online sessions over an extended period of time was difficult due to the lack of social connectedness between co-design participants [32]. Also, the clinicians, in our study, reported the limited efficacy of virtual communication, which perhaps reflects the widely experienced videoconferencing fatigue experienced by professionals in health care and other similar service domains [33,34]. Together, these results indicate the need to: identify the types of co-design discussions that suit virtual or face-to-face settings; improve the design and processes around videoconferencing to make it a more acceptable medium for co-design discussions; and reintroduce face-to-face co-design sessions as we transition back to the “new-norm.”

The challenges we identified with the preimplementation processes appeared to have a lasting impact on health IT outcomes as they contributed to poor clinician satisfaction and a negative patient experience. For example, in the co-design process, participants from the IT team reported that new work requiring UX expertise was added to their key tasks during the design and implementation of health ITs during the pandemic, thus changing their roles and responsibilities. As their work was previously focused on configuring health ITs from vendors rather than co-designing health ITs with users, the implemented health ITs were sometimes seen to be unsatisfactory to the users. This draws attention to the need for health care organizations to review their IT staff skills and capabilities and align them to the capability needs of a proposed health IT project so that existing staff members are not required to learn and apply new skills to health IT projects simultaneously. Furthermore, it is imperative for organizations to analyze existing and proposed processes (eg, co-design process) systematically at the organizational level as health IT implementations inevitably lead to new work and redistribution of existing work [35,36]. Consequently, it is imperative for health care organizations to ensure health IT implementation processes cater for work changes within and across clinicians and IT teams rather than the mere purchase of health ITs [37].

In addition to communication and process improvements highlighted in this study, our findings revealed digital health workforce development needs for both clinical and IT students and professionals, to address knowledge gaps and communication challenges. For clinicians, digital competence is typically viewed as knowledge, skills and motivation to use and interact with technologies [38-41]. However, we contend that the perception of *clinicians as users* [42] of digital health technologies must evolve to that of a continuum where clinicians can be at any point of the spectrum that *clinicians as users* to *clinicians as co-designers*. We propose that digital health curriculum should offer clinicians an opportunity to learn the principles of HIT co-design, and the health IT design, development, and implementation lifecycle, to ensure clinicians are equipped with the capabilities and skills to both practice in digital environments and drive digital health innovations. In addition, the suboptimal patient experience reported in our study highlights the need to ensure that patients are also involved as users and co-designers of patient-facing health ITs.

Whilst significant attention has been placed on the development of digital competencies for clinical students, the development of health competencies for IT professionals designing, developing, and implementing health ITs in clinical settings is not as advanced [43-45]. Although clinical informatics programs attempt to bridge this gap, it is not a requirement for IT professionals working in clinical settings, and these skills are sometimes learned on the job [46]. Our findings suggest that IT professionals working in clinical settings may need additional training in the “health sciences” domain, which could encompass knowledge in areas like health concepts, and health sector structures and roles [47].

### Strengths, Limitations, and Future Directions

The results from this study have made contributions to the research, practice, and educational requirements for health IT implementations. From a research perspective, we have adapted the interdisciplinary team communication framework (Figures 1-3), incorporating the macro-environmental factor (COVID-19 pandemic) that has significantly impacted health IT implementations since 2020. We tailored the framework to unpack complex structures, processes, and outcomes involved in pandemic-driven health IT implementations between teams. The adapted framework will be useful for researchers undertaking process evaluations for health IT implementations that have occurred since the pandemic.

From a practice perspective, we identified communication structures, including the medium and approach, as key factors that impact health IT implementation processes and outcomes. In our setting, this study resulted in a review of the communication structures available to facilitate better development of interactions and connections that exist within and across internal and external teams. It also led to concrete steps being taken to ensure face-to-face meetings occurred, the creation of office space for IT staff to co-locate in the virtual hospital, and the sustainability of the clinical informatics expertise to work with both the clinician and IT teams.

From the education perspective, we have brought to light the importance of ensuring that digital health education accommodates clinicians as they transition from users to co-designers of health ITs. We further identified the need to better incorporate “health science” knowledge domains in IT curriculum [45] for IT professionals who deliver HITs in clinical settings.

Limitations of our study include that it was an exploratory study that focused on 2 teams within a health service, which may limit the generalizability of the results. The adapted interdisciplinary framework for HIT implementations during the pandemic needs to be validated and studied in the context of other team-based settings. Also, as patients were unable to be recruited for this process evaluation study, our findings may not adequately capture patient perceptions of health ITs during the pandemic. Future work will involve determining the extent to which findings from this study transfer to brick-and-mortar hospital settings and in other interdisciplinary team settings.

### Conclusion

The primary purpose of health IT is to facilitate the delivery of quality health care and improve health outcomes [48], but these can only be realized if health IT implementations are successful and health IT is used. Various technological, people, organizational, and macroenvironmental factors have impacted health IT implementation success, more so since the COVID-19 pandemic. Specifically, we found that team structures, communication pathways, and health IT preimplementation processes (project management and co-design) were negatively impacted by the pandemic. While health service organizations are keen to transition to the “new norm,” it is imperative to learn from the health IT implementation successes and failures that occurred during the pandemic via process evaluations. Our process evaluation offers learnings for the research (an adapted interdisciplinary team communication framework), practice (the need for health care organizations to review their communication structures, IT staff skills, and proposed processes), and educational requirements (need for better education and training of IT professionals working in clinical settings on health concepts) of health IT implementations as the world transitions to the “new norm.”

## Appendix

Multimedia Appendix 1 COREQ (Consolidated Criteria for Reporting Qualitative Research) checklist.

Multimedia Appendix 2 Interview Guide.

## Abbreviations

COREQ: Consolidated Criteria for Reporting Qualitative Research
TPOM: Technology, People, Organizations, and Macro-environmental factors
UX: user experience

## Data Availability

The datasets generated and analyzed during this study are not publicly available as per ethics approval.
