# Supplementary material for: Health IT Implementation and the Impact of the COVID-19 Pandemic on Clinician-IT Dynamics: Qualitative Study
Source: J Med Internet Res. 2025 Feb 11;27:e57847. doi: 10.2196/57847 (PMC11862760; doi:10.2196/57847)
Supplement: Multimedia Appendix 2 [file jmir_v27i1e57847_app2.docx]

**Supplementary files_ Appendix A**

**Manuscript**: A process evaluation of health information technology implementation: Impact of the COVID-19 pandemic on clinician-IT dynamics

**Interview guide**

Thank you very much for agreeing to participate in this interview.

The purpose of this session is to understand the process of rapid design, development and implementation of technology at [the virtual hospital]. I’ll ask some questions to guide the discussion and keep us on track, but feel free to speak freely and openly as we’d like to hear your views.

The session will be audio-recorded and then transcribed. Any identifiable information will be removed during transcribing, and de-identified transcripts will be analysed. Audio-files will then be deleted. This means that no identifiable data from you will be used in the analysis or any output from this interview.

Are there any questions before we start?

Is it okay if I ask you a few basic demographic questions before we start?

1. What is your role at [the virtual hospital]?
2. How long have you been in this role?
3. How many years of clinical [or ICT] experience do you have in total?
4. Are you currently a full time, part time or casual staff?
5. Can you please let me know what gender you identify as?
6. May I know your age or age range please?
7. In a couple of sentences, can you describe what you do at [the virtual hospital]?

Thank you for answering those quick questions. We will now proceed to the core interview questions.

**General overview**

1. What is your overall impression of [the virtual hospital]? (Prompt: technology, clinical care, governance, workplace culture)
   1. What works well and what doesn’t work so well?

**User perception of technologies at [the virtual hospital]**

1. What about the technologies you currently use (or have used) at [the virtual hospital]? Can you describe the key technologies you use and how?

**The technologies**

1. How would you rate the performance and dependability of the key technologies you’ve just described? [Prompt]
   1. Are they easy to use? Acceptable to all end users?
   2. Any problems?
   3. Did the technologies perform at the level you expected them to?
   4. What value do these technologies add to the service and care delivered by [the virtual hospital]?
   5. Do the technologies deliver the benefits you expected them to? Why or why not?

**Perception of the health information technology implementation process**

1. Tell me about the rapid development of remote monitoring technologies at [the virtual hospital]? *[If new, ask about expectations re how it should have been designed and developed]* [prompts]
   1. Can you describe how these technologies were designed and developed?
   2. Did this process align with your expectations of how technologies should be designed and developed for a clinical service? Why or why not*? [IT only: What about the expectations of clinicians? Are your expectations consistent with those of clinicians?]*
   3. Do you think it’s been done well so far? Why or why not?
   4. What worked well and didn’t work well?
2. What about implementation? What process is used for rolling out new technologies? *[If new, ask about expectations re how it should have been designed and developed]*
   1. Do you think implementation has been done well so far? Why or why not?
   2. Is this aligned with your expectations of how the technologies should be implemented? Why or why not? *[IT only: What about the expectations of clinicians? Are your expectations consistent with those of clinicians?]*
   3. What worked well and didn’t work well?
3. Can you think of any improvements needed to the way new technologies are developed or implemented in [the virtual hospital]? *[If new, ask about the ideal way new technologies should be developed and implemented*] What would you change going forward?

**People**

1. Can you think of any barriers or hurdles to ongoing collaboration with other stakeholders such as ICT services, product developers, other clinicians to design new digital solutions for [the virtual hospital]? [prompt]: trust

**The organisation**

1. How would you rate [the virtual hospital’s] overall capacity to:
   1. Participate in the design of digital solutions? Why?
   2. Implement new digital solutions? Why?
2. Is this different to traditional hospitals, eg [brick and mortal hospital], in general?
3. To what extent do you think [virtual hospital’s] routines, pathways and processes will need to change to accommodate new technologies?
